# Assistance for parents with unsettled infants in Central Vietnam: a qualitative investigation of health professionals’ perspectives

**DOI:** 10.1186/s12887-019-1532-5

**Published:** 2019-05-20

**Authors:** Linda Murray, Thach Tran, Vo Van Thang, Nicole McDonald, Sean Beggs, Jane Fisher

**Affiliations:** 1 School of Medicine, University of Tasmania, Hobart, Tasmania Australia; 2 School of Public Health and Preventive Medicine, Monash University, Melbourne, VIC Australia; 3 Institute of Community Health Research, Faculty of Public Health, College of Medicine and Pharmacy, Hue University, Hue, Vietnam; 4 School of Public Health and Social Work, Queensland University of Technology, Brisbane, QLD Australia; 5 College of Health Sciences, Massey University, Wellington, New Zealand

**Keywords:** Infant sleep, Settling, Breastfeeding, Colic, Vietnam, Health education

## Abstract

**Background:**

Unsettled infant behaviours are a common concern for parents internationally, and have been associated with maternal stress, reduced parenting confidence, and postnatal mental health problems among parents. Little information currently exists regarding available support for the parents of unsettled infants in low-and-middle income countries such as Vietnam. We aimed to describe how unsettled infant behaviour was understood and investigated by Vietnamese health professionals, and what health education was provided to parents regarding infant sleep and settling.

**Methods:**

This qualitative study elicited the perspectives of Vietnamese health professionals working in Thua Thien Hue Province, Vietnam. A semi-structured interview guide included participant demographics, and questions about providing assistance to the parents of unsettled infants, understandings of unsettled infant behaviour, management of unsettled infant behaviour and health education. Individual interviews or small-group discussions were undertaken in Vietnamese, data were translated and analysed in English. The authors used a thematic approach to analysis, supported by Nvivo software.

**Results:**

Nine health professionals (four primary care doctors, one paediatrician and four nurses/midwives) working in urban and rural areas of Thua Thien Hue were interviewed. Four themes were created that reflected the responses to the literature-based interview questions. Health professionals described having received little formal training about infant sleep and settling, thus based their advice on personal experience. Information on infant sleep and settling was not included in health education for new mothers, which focused on breastfeeding and preventing malnutrition. Where advice was given, it was generally based on settling strategies involving high levels of caregiver intervention (holding, rocking, breastfeeding on demand and tolerating frequent overnight wakings) rather than behaviour management style strategies. Participants emphasised the importance of recognising and responding to infant behavioural cues (e.g infants cry when hungry).

**Conclusions:**

There is an unmet need for information on infant sleep and settling for new parents and health professionals in Vietnam. Our findings suggest information for caregivers on how to respond sensitively to infant tired signs should be formally included in the training of health professionals in LALMI settings. Sleep and settling information should also be part of culturally appropriate multi-component maternal and child health interventions aimed at promoting early childhood development.

**Electronic supplementary material:**

The online version of this article (10.1186/s12887-019-1532-5) contains supplementary material, which is available to authorized users.

## Background

Unsettled behaviours among infants (babies aged 0–12 months) are defined as behaviours that include excessive crying episodes, being inconsolable and unreceptive to soothing, difficulties falling asleep, brief durations of sleep, and frequent waking periods during the night [1]. Amongst afebrile infants, a physical cause for unsettled behaviours (e.g. hunger, gastrointestinal reflux or allergies) can only be found in around 5 % of cases [1–3]. Among infants who are otherwise well, unsettled behaviours remain difficult to explain, but are thought to be multifactorially determined. Factors such as infant temperament, and parents’ caregiving practices and sleeping arrangements have each been associated with infant sleep problems and total daily duration of crying [1, 4].

Multiple terms have been used to describe unsettled infant behaviour including colic, excessive crying, fussing and infant irritability [1, 5]. In the 1950’s, Wessel and colleagues introduced the “rule of three” to define colic as occurring among infants who cried for at least three hours per day on at least three days of at least three consecutive weeks [6]. Whilst different definitions exist, and the term is understood differently by lay people and professionals, colic is generally used to describe healthy infants who inexplicably and inconsolably cry for prolonged periods [7]. As there are variations in the use of the term ‘colic’, identifying and defining specific infant behaviours that parents seek help for may be more useful than such generalised terms. More recently, in the United State and Canada, the “period of PURPLE crying” concept has been used to describe the period between 2 week and 3–5 months of age where an infant may inconsolably cry for increasing periods each day, reaching a peak at around the second month of age [8]. This concept has been used to educate parents about understanding and responding to infant crying and to raise awareness of the risks posed by prolonged crying for maltreatment of infants including shaken baby syndrome [9].

Evidence from high-income countries indicates that unsettled infant behaviours are one of the most common reasons that parents seek assistance from health professionals [10, 11]. Despite problem being common, the way in which unsettled infant behaviours are understood, investigated and managed by health professionals is inconsistent. Conflicting or inconsistent advice from health professionals about the causes, and effective responses to unsettled infant behaviour is confusing for new parents, who may already be experiencing feelings of stress and under-confidence. Mothers with infants who cry excessively report significantly higher parenting stress and lower feelings of efficacy than the mothers of infants without excessive crying [12]. In high-income countries, clinical depression rates are approximately twice as prevalent amongst mothers with unsettled infants [13, 14]. Whilst most evidence of this association originates from cross-sectional studies, meaning the direction of the association cannot be determined, two longitudinal studies have demonstrated that excessive inconsolable infant crying preceded postnatal depressive symptoms [15, 16]. There is also evidence that mothers’ perceptions of their ability to soothe their infant may be more relevant to postpartum depressive symptoms than crying duration alone [16].

Perinatal common mental disorders (PCMDs) are predominantly socially determined and are often correlated with unsettled infant behaviour [17, 18]. In LALMI settings, PCMDs have been associated with socio-economic disadvantage, experiences of intimate partner violence, low partner empathy or support, and insufficient emotional and practical support [17]. Whilst there is evidence that unsettled infant behaviour is associated with PCMDs in high-income settings [13–16], the prevalence of unsettled infant behaviour and sleep disturbance in low and lower-middle income (LALMI) countries is rarely reported. In a large cross-sectional study, Sadeh et al. [19] identified a higher prevalence of parent reported sleep problems in “predominantly-Asian” countries (52%) compared to “predominantly-Caucasian” countries (26%), but did not analyse differences between LALMI and high and upper-middle income Asian countries.

Little is known about how health professionals in LALMI settings such as Vietnam conceptualise unsettled infant behaviour, and what professional advice and support they offer to the families of unsettled infants. This study aimed to explore how unsettled behaviour was understood, clinically investigated and responded to by Vietnamese health professionals, and to describe what health education on infant sleep and settling was available.

## Methods

A deductive qualitative methodology was used to elicit data about the perspectives of Vietnamese health professionals in order to describe how unsettled infant behaviour was understood and managed, and to describe what education was available to parents. The question guide design was informed by a review of existing literature on unsettled infant behaviour and responses in high-income countries, and where available, LALMI settings [1, 17] (see Additional file 1). Data were collected between December 2014 and February 2015.

## Setting

Thua Thien Hue Province, Central Vietnam has a population of approximately 1,150,000 people who live in Hue City and eight rural districts [20]. Hue city was the ancient imperial capital of Vietnam, and is an important site for Vietnamese Buddhism. Thua Thien Hue Province has two hospitals that provide tertiary health care for children. Primary and secondary health care services are available through a network of commune health centres and district hospitals.

### Participants and recruitment process

A purposive sampling strategy was used to recruit health professionals from urban and rural health services. Health professionals who worked in clinical settings where parents with unsettled infants may seek help were considered eligible for recruitment. This included midwives and general doctors working in community health centres that provided child health checks and services, and child health specialists working in tertiary settings (e.g paediatricians). All health professionals approached agreed to be interviewed.

### Data sources

A question guide was developed based on a review of current evidence about unsettled infant behaviour and parent education in both high-income and LALMI settings and investigators’ expertise and experience. The question guide began with structured questions on demographic information (occupation, gender, location of clinical work (urban/rural) and years of experience). The rest of the question guide involved semi-structured, open-ended questions under four main domains: Experiences of assisting with unsettled infant behaviour (how often parents with an unsettled infant presented to their health service); understandings of unsettled infant behaviour (how professionals understood the causes of unsettled infant behaviour, what professional training they had received); strategies for managing unsettled infant behaviour (strategies used to investigate, treat, and advise parents about unsettled infant behaviour); and education (whether their health service provided education on infant sleep and settling to new parents). The question guide was translated from English to Vietnamese and checked by two bilingual research assistants for comprehensibility, and cultural acceptability.

### Procedures

Individual interviews or small group discussions took place in participants’ workplaces and were recorded on an MP3 recorder. Interviews were conducted by an English-speaking researcher and were contemporaneously interpreted by a bilingual Vietnamese research assistant with university level qualifications in English. Contemporaneous interpretation is considered optimal for conducting qualitative research in multiple languages as it allows points of confusion to be clarified at the time of interview [21]. The English interpretation from recordings were transcribed, and another translator with postgraduate qualifications in English reviewed the recordings and transcripts to verify the accuracy of the interpretation.

### Ethics

Human Research Ethics Committees in Australia (University of Tasmania: approval no H001440), and Vietnam (Hue University of Medicine and Pharmacy: approved October 1, 2014) approved this research.

### Data management and analysis

The translated transcripts were analysed by members of the research team [LM, TT, TV, JF] using Nvivo (version 10) software for data management. Theoretical thematic analysis was undertaken using the principles of thematic analysis outlined by Braun and Clarke [22]. Thematic analysis was driven by the researchers’ theoretical area of interest rather than by approaching analysis without preconceived notions of what the data contained. As discussed by Braun and Clarke (p 10) a group of quotations was considered a theme once researchers agreed the theme captured something important about the data in relation to the research questions, and represented some level of patterned response within the dataset [22]. The aim of thematic analysis was to produce themes that described the socio-cultural and professional contexts that informed the individual accounts provided within the data [22, 23].

## Results

Overall 9 health professionals were recruited from the following professions: Primary care doctors (*n* = 4), midwives/community nurses (n = 4) and paediatricians (*n* = 1). Most participants worked in commune health centres (6 urban/2 rural) and one worked at a tertiary hospital. Participants all had over ten years of professional experience, and seven were female. Seven individual interviews took place and one small group discussion (*n* = 2). Primary care doctors and community nurses at commune health centres generally only routinely checked on babies until around six weeks of age, although all participants saw children until at least one year of age if they were brought in for consultation. Participant interviews provided a rich dataset that resulted in four final themes after coding using Nvivo.

### Sources of support for the families of unsettled infants

Health professionals informed us that no specialised services for women with unsettled infants (of any age group) existed. If new mothers were exhausted or babies cried excessively, extended family members provided assistance in the first instance:

*“Not here (in Hue), at this moment we don’t have any kind of support where you have a place where the mother can bring the baby and receive advice to solve this situation. Normally you stay at home and the families have to take care of the baby together. If the mother is too tired, then the father has to be there, or the mother in-law, or maybe a nanny.”* – Paediatrician, female, tertiary hospital

*“(if the baby is crying excessively) She (the mother) should go to a hospital or the central hospital to find the reason for the crying. In some cases it’s crying but it’s normal, so the family manage it themselves.”* Primary care doctor, female, urban CHC

If parents did access health services, concerns about unsettled infants were addressed within a primary health care model together with feeding and other child health problems. Unsettled infant behaviour was a common reason mothers presented to the commune health centre (CHC) and study participants described feeling like they were the only source of professional support available to these parents:

*“The mother usually takes some time to decide (if the child is) very hard to get to sleep and also crying, and they would like to find out the cause. Babies that are hard to get to sleep and also crying are one of the common reasons they (mothers) bring the baby to the CHC.”-* Primary care doctor, female, urban CHC

*“Because the mother maybe does not have enough milk so the baby often cries a lot and the mother also cries, and the grandmother also cries. And I have to sit with them all night. I am the only midwife here, and I have to do all of it.”* Midwife, female – rural CHC

#### Other forms of support

Several participants mentioned that when no cause could be found for unsettled infant behaviour, especially in young infants (three months and under) parents might seek help at Buddhist pagodas. It was explained that Buddhist monks could perform ceremonies (such as saying prayers/chanting) intended to stop excessive crying, and that parents believed such ceremonies were effective:

*“When the baby cried a lot but they didn’t find any reason why the children cry, and after that they took him to the pagoda…It’s hard to believe that, but here it is the perception of the new parents. People believe that.”* Primary care doctor, male, rural CHC

It was explained that this practice was not endorsed by health professionals, but was seen as a last resort and worth trying because it was unlikely to cause harm:

*“Here (Hue) is a centre of Buddhism. We have a strong relationship with the pagoda, because you know it’s a no-harm solution, you just go there. Even the young generation, the young parents don’t believe the religion as much, but because it doesn’t cause harm, and maybe a spiritual matter, they just go there.”* – Paediatrician, female, Tertiary hospital

### Understandings of unsettled infant behaviour

#### Describing crying as “normal”

Participants were asked to describe what they thought caused unsettled infant behaviour. We also enquired if they were familiar with the concept of “colic”. No participants were familiar with this term and no Vietnamese equivalent existed. However, some professionals did advise parents of infants up to three months of age that a certain amount of crying in well infants is “normal”. Some participants referred to a Vietnamese saying about babies crying for “three months and ten days”:

*“Sometimes the baby cries and the mother cries, because they can’t find any way to settle them…We just advise them that it is normal, it’s a stage of the baby’s development and we ask them to feed the baby and weigh the baby.” Midwife, female, Rural CHC*

“*First, the mother should be monitoring the children’s weight and development. If it is normal then I just give advice…There is a tradition that 3 months and 10 days it is the special time for the mother, and the children cry a lot, and after that they will stop*.” Paediatrician, female, Tertiary Hospital

All health professionals stated that when they encountered a mother with an unsettled infant, their first action was to look for a physical cause relating to illness or malnutrition, and to assess the child’s developmental milestones:

*“There are two things we recommend for the mother. The first one is to check the weight of the children. When monitoring the weight, if it is normal, then maybe the child will stop. If they are still crying, parents should go to the CHC to check with the specialist and get a clinical examination. If there is the potential for a health problem they should explore that.” Doctor, female, urban CHC*

*“I would like to ask about the reason the baby cries. First of all, does the baby have an illness? Or is it hungry? Or is the mother not feeding it enough from the breast?” Doctor, male, urban CHC*

*“First, I will conduct a health examination for the baby. Or teach the mother about appropriate child development depending on their month of age. And after the children go home, the mother may notice some more issues, like if the children aren’t paying attention, or they aren’t smiling, or if they don’t try to communicate with the mother. If the children don’t do that, parents should be aware that there could be a problem…If after two months the children don’t do anything or they aren’t “talking”, then they should go to the CHC, because maybe the children has developmental problems.”* Primary care doctor, female, Urban CHC

No participants mentioned overstimulation or overtiredness as possible reasons for unsettled behaviour in infants and young children. If no illness or evidence of malnutrition or developmental delay was found, parents were offered a range of general advice on infant sleep, which is summarised in the next theme.

### Advice given to the families of unsettled infants

Participants stated that advice given to parents regarding unsettled behaviour in otherwise well infants was only provided if requested, and usually focused on interpreting infant behavioural cues. Many health professionals suggested that mothers had low health literacy, and if they were first time parents, needed assistance learning how to recognise when their infant was hungry or required a nappy change, and how to respond appropriately. It is unclear whether participants explained that the cause of infant crying is often indistinguishable, and therefore parents must also pay attention to the context of the crying. Participants also recommended that parents learn to bring their infant to the health centre if they suspected they were unwell.

*“We often just advise the mother (on their situation) because their knowledge is very low”. Doctor, male, urban CHC*

*“If the children usually wake up at night and cry a lot without any reason, we’ll suggest the mothers should check that if the child is hungry or they are cold in the winter or hot in the summer. The mothers should find out the reasons that make their child irritated... If the child is hungry, we have to do nothing but feed him. Some reasons for crying include starving, feeling hot or cold feeling or wet. This is my experience I share with the mums.” Midwife, female, urban CHC*

When such cue-based care was recommended, it involved advice to let babies under six months old feed and sleep “on-demand”. Participants did not mention behavioural interventions such as feed-play-sleep programs, or any other specific advice for infants older than six months of age:

*“For the new baby they often sleep all day time but they are awake at night time so the mother has to stay awake with them, and then maybe at night they are always hungry, so you must feed them.” Midwife, female, rural CHC*

*“We don’t give the mother direct guidelines, however I think that the baby should be sleeping at the children’s demand. For example, some children want to sleep during the day, but at night they wake up and they play.”* Primary care doctor, male, rural CHC

#### Providing advice from their own experience

None of the participants in this study had ever received any formal training on infant sleep and settling for children of any age to apply to their professional practice. However, some health professionals mentioned that they had given specific advice in relation to settling infants based on their “own experience” as either clinicians or parents:

*“For some skills, like weighing the baby, I got some training. But for advising the mother on psychology or sleep, it’s just my experience from many cases.”* Paediatrician, female, tertiary hospital

*“In the community so many people come here to ask advice… I am a parent and I have children, so I can speak from my experience (of being a parent).”* Doctor, male, rural CHC

This advice included teaching mothers how to hold or position their baby so they may be comfortable, and telling mothers to try to be “less stressed”:

*“When babies cry a lot, the mother should calm down and not be nervous, just let the baby to be more comfortable, and this will calm the baby and they won’t cry any more.”* Midwife, female, urban CHC

*“I tell them that maybe the baby just wants to hug, be close, there are many shapes (positions for holding the baby) and I also check about breastfeeding”* Male doctor, rural CHC

### Education about infant sleep and settling

All the professionals interviewed described conducting health education for pregnant women and new mothers (up to six weeks postpartum), either through group education sessions or during individual consultations. All participants stated that standard information on infant sleep and settling was not routinely provided to women through such health education activities. Rather, the health information presented focused on the prevention of malnutrition through promoting exclusive breastfeeding for the first six months of life followed by the introduction of appropriate complementary foods:

*“We don’t talk about that (sleep and settling), we are mainly focussed on the topic of breastfeeding and nutrition, how the mother can cook nutritious food.”* Midwife, female, rural CHC

*“We sometimes do this (provide settling advice) but it’s not usual, especially not in the program where we weigh the children to evaluate which ones have malnutrition.”* Primary care doctor, male, rural CHC

The focus on nutrition information was attributed to the historically high rates of child malnutrition in the area:

*“Ten years ago there was a lot of malnutrition here. However because of recent economic development the nutrition is better for the baby, so malnutrition is not a big problem any more, except for special cases like the preterm baby or a child with chronic disease.” –* Paediatrician, female, tertiary hospital

*“Regarding some cases of how to settle a baby when they cry a lot, first find the cause for why the child is crying because it could be things like recent immunisation, or they may need medication or to go to a specialist doctor, or the mother may need some “psychology”. It could also be practical, like how to hold the baby, because every 3 months I go to visit the household to weigh babies 0–6 months old and to examine children that may have malnutrition, and refer cases with problems to the CHC or the doctor. I also find out some of the issues behind unsettled behaviour like infant illness or the economics or the education of the parents. If parents have low education I can teach them: babies should be held close, when the children have diarrhoea I can teach the mothers hygiene, or something like that.” – Midwife, female, rural CHC*

## Discussion

This study is the first to explore how Vietnamese health professionals conceptualise and manage unsettled infant behaviour, and what health information on sleep and settling is available to Vietnamese parents. The findings revealed that unsettled infant behaviours are a serious, recognised and complex problem in Vietnam and potentially therefore in other LAMI countries. Health professionals in this study stated that infant settling advice was given by informal sources such monks at Buddhist pagodas. This was seen as a ‘last resort’ when other sources of advice such as family members and health professionals failed to meet the needs of parents with unsettled infants. It is known that the caregivers of unsettled infants in Vietnam consult sources such as family members, buddhist monks and other types of traditional healers before presenting at formal health services [24]. In the absence of health professional training in infant sleep and settling, little effective, evidence-based advice was available for the parents of infants, leaving them to rely on informal approaches without a literature supporting their effectiveness. These findings are significant as there are known negative consequences for the caregivers of unsettled infants (such as decreased confidence and increased stress), as well as for the caregiver-infant relationship [12, 13].

Strengths of this study included the use of qualitative methodology to elicit the perspectives of health professionals in light of their sociocultural and professional context. Participants were interviewed in their own language, which allowed them to express concepts and insights in a more comfortable way, resulting in a richer and more nuanced dataset. Participants from both urban and rural areas were included in order to provide a representative sample of health professionals who work with children throughout the province.

In terms of limitations, the inclusion of a broader range of health professionals, such as those working in different tertiary settings, might have resulted in a wider range of perspectives. Due to the Principal researcher not speaking Vietnamese, interpreters were required for most interviews, which can lead to errors of translation. Despite this, use of contemporaneous interpretation and independent verification of all interview transcripts ensured rigour of translation [21]. The results of qualitative studies are not generalizable to other settings, where there may be differences in health service provision, health professional training, and the education provided to new parents. Therefore, the findings of our study are relevant only in the Vietnamese context.

No health professionals had received formal training on unsettled infant behaviour as part of their professional qualification, and hence based their advice to parents on personal experience. This finding is not unexpected as Mindell et al. [25] found that internationally, content about sleep in paediatric residency programs is minimal, and in Vietnam, paediatric programs contain no information on normal sleep, psychology or behavioural insomnia in childhood. When conceptualising possible causes for physically unexplained unsettled infant behaviour, no Vietnamese health professionals used the concept of “colic” or the “period of PURPLE crying”. Rather, insufficient breast milk was perceived to cause excessive crying. This is consistent with published evidence that reports ‘hunger’ is the most commonly assumed reason for unsettled infant behaviour in both high-income and low-and-middle income settings [26–28]. Participants also stated that they advised parents that crying in early infancy was ‘normal’ and would cease after three months and ten days. Interestingly, this advice does correspond with evidence that healthy infant crying reaches a peak at around two to three months of age and then declines, which is also taught as part of the period of “PURPLE” crying concept in the USA and Canada [8, 29]. In this study, no participants referred to standardised advice for settling infants of different ages that was provided by health services.

The explanations for causes of unsettled infant behaviour that were given by health professionals were based around the mother not understanding and responding to infant cues. Responding sensitively to infant cues is internationally recognised as an important part of providing nurturing care for infants to promote optimal health outcomes [1, 30]. Previous qualitative research has revealed that Vietnamese mothers and grandmothers received settling advice solely from other family members, and this advice focused on interpreting unsettled infant behaviour as “loneliness” or hunger [28]. There is currently no published research regarding Vietnamese caregiver’s expectations of infant behaviour (e.g duration of crying) or sleep patterns at different ages, nor ethnographic research about how cultural practices such as postnatal confinement influence caregiver settling techniques. Such research is recommended as it would provide insight into what advice and interventions are likely to be effective and socially acceptable in this context.

In high-income countries such as Australia, advice regarding the causes and appropriate responses to unsettled infant behaviour appears to exist on a spectrum between two main positions: the “intuitive parent” position and the “infant behaviour management” position. The “intuitive parent” position is based on the premise that parents should follow their “intuition” about their babies’ needs as opposed to a strict set of guidelines about when to feed, settle or respond to a baby’s cry [31, 32]. Advocates of this position suggest interpreting and responding to infant cries with active comforting techniques (such as rocking and walking) and frequent overnight waking, co-sleeping and feeding to sleep. Alternatively, the infant behaviour management position recognises that unsettled infant behaviour can cause significant problems for some families, and once organic illness is ruled out, a cause may not be identifiable [1]. This position assumes that if parents are seeking help regarding unsettled infant behaviour, it is likely the baby is crying for longer and more intensely than the average baby, and that this is contributing to poor family functioning [33]. Infant behaviour management approaches assert that parents can acquire knowledge and skills about their baby’s developmental capacities, which can be translated into settling strategies to assist their babies to self-soothe and return to sleep independently when they wake up [1]. There is agreement on some aspects of unsettled infant behaviour between the two positions, including: trying to enhance pleasurable interactions between infants and parents; promoting sensitivity and responsiveness to infant cues; acknowledgement that healthy babies under six months will need their parents for feeding and help to settle during the night; that infant crying is care-eliciting behaviour, and that a baby should never be shaken [1, 34, 35].

The advice health professionals in Vietnam gave regarding how to respond to unsettled behaviour tended to align more with the ‘intuitive parenting’ position. This included participants suggesting interventions with high levels of caregiver involvement such as constantly holding and patting babies and feeding them on demand. However, it should be noted that not all participants differentiated whether they gave advice according to the age of the infant. In the commune health centre context, infants are only routinely seen up to three months of age, although infants of any age can be brought in for consultations. None of the participants recommended advice on infant settling strategies linked to a scientific evidence base, or that referenced the infant behaviour management position or “sleep training” style programs or advice for older infants [1]. It therefore appears that this approach was not familiar amongst Vietnamese health professionals.

Participants described how health education and primary health care programs for pregnant women and new parents were heavily focused on breastfeeding and did not include information on infant sleep and settling. There is a historical and political context as to why this is the case, as infant and child malnutrition was very common in Vietnam, especially during and immediately after the war from 1955 to 1975. However, over the last decade, rapid economic development, and public health programs to addressed micronutrient deficiencies have reduced malnutrition in the majority Kinh population [36]. Despite a substantial decrease in malnutrition rates at a population level, many Vietnamese children under five years of age (particularly ethnic minorities and the socioeconomically disadvantaged) still experience malnutrition (40% underweight, 36% stunting and 10% wasting) [36–38]. Vietnam also has low rates of exclusive breastfeeding (feeding only breast milk until six months of age) at around 20%, and Thu et al. [39] suggest the benefits of exclusive breastfeeding are not well understood by Vietnamese women [39, 40]. Therefore the health education provided by primary health professionals was generally focussed on infant and child nutrition. Whilst this focus is undoubtedly important, it has been noted that internationally, the provision of health education on infant sleep and settling has been given less priority in health education [1].

Postnatal common mental disorders among women are prevalent in low and lower-middle income countries, and up to one-third of women in Vietnam experience a postnatal PCMD [17, 41, 42]. One cross-sectional study identified an association between prolonged infant crying and higher Edinburgh Postnatal Depression Scores in Ho Chi Minh City [41]. There is also a documented correlation between malnutrition amongst the children of mothers with PCMDs in Vietnam and other LALMI settings [41–44]. Qualitative research reveals that the mothers of infants up to six months old in Vietnam experienced feelings of anxiety, helplessness, being overwhelmed, and a loss of control when their infant cried excessively [24]. As PCMDs occur commonly in Vietnam, but are currently an under-recognised health concern, improving the understanding and management of infants with sleep issues and excessive crying could have positive impacts on PCMDs in Vietnam. Whilst there is general consensus that infants under six months of age will need assistance to settle and wake for feeds overnight, there is some evidence that programs that assist older infants to self-settle can decrease the frequency of night wakings, which may assist family functioning if caregivers are also getting more sleep. Therefore training health professionals to provide information on normal infant sleep patterns at different ages, and culturally appropriate settling strategies for infants over 6 months could be helpful additions to interventions aimed at assisting children to survive and thrive.

It is recommended that infant sleep and settling advice be included as part of multi-component interventions that encompass the child, primary caregivers and the relationship between caregivers and children. Such multi-component interventions align with the World Health Organisation “nurturing care” framework as part of the focus on early childhood development of the 2030 Sustainable Development Goals [30]. As part of this framework, interventions that include support for caregivers to provide ‘nurturing care’ (a stable environment that includes protection from threats, opportunities for early learning and affectionate interactions and relationships) is required in addition to promoting optimal health and nutrition [45]. In Vietnam, nurturing care education and interventions should consider the cultural context regarding current family sleeping arrangements, postnatal confinement practices, the inclusion of multi-generational caregivers, and caregiver expectations of “normal” infant behaviour and sleep patterns.

## Conclusions

The findings of this study suggest that there is little evidence-based information on infant sleep and settling included in health professional training, or provided to new parents in Vietnam. We recommend that information on understanding age appropriate infant sleep patterns and signs of tiredness, responding to infant crying and infant settling techniques should be included in maternal and child health education in Vietnam as part of multi-component interventions for caregivers that promote “nurturing care”. It is also recommended that further research into settling practices and caregiver expectations about infant sleep and crying be conducted in Vietnam, so health professionals who provide advice on infant sleep and settling can ensure interventions are supportive and culturally acceptable.

## Additional file

Additional file 1: Appendix B: Question Guides. (DOCX 19 kb)

## Abbreviations

LALMI: Low and Lower-Middle Income
PCMD: Perinatal Common Mental Disorder
Period of PURPLE: Period of “Peak” (of crying), “Unexpected” (crying), “Resists” (soothing), “Pain-like” Face, “Long” lasting, “Evening” (crying)
USA: United States of America
