# Supplementary material for: Assistance for parents with unsettled infants in Central Vietnam: a qualitative investigation of health professionals’ perspectives
Source: BMC Pediatr. 2019 May 20;19:160. doi: 10.1186/s12887-019-1532-5 (PMC6526599; doi:10.1186/s12887-019-1532-5)
Supplement: Supplementary file 1 — Appendix B: Question Guides. (DOCX 19 kb) [file 12887_2019_1532_MOESM1_ESM.docx]

**Question Guide for Health Professionals: Understanding and Responding to crying and sleep problems in infants in Central Vietnam: A qualitative investigation**

Years in practice:

Profession:

Location of work (urban/rural):

Male/Female:

1. Do you ever see people who have problems with their baby crying or waking frequently?

1. What criteria would you use to consider excessive crying in a new infant as a health problem?
2. Are there particular words or diagnoses commonly given to infants who cry excessively?
3. What treatments or management would you recommend for this?
4. What strategies do you think new mothers and fathers use to settle their infants?
5. What advice do you give new parents about infant sleeping and settling?
6. What do you teach new mothers about:

- infant settling?
- infant cues?
- maternal self-care?
- feeding/sleep cycle?

9. Is there any other advice you could give us about how excessive infant crying is managed in Vietnam?
